# Substrate Specificity of Chimeric Enzymes Formed by Interchange of the Catalytic and Specificity Domains of the 5′-Nucleotidase UshA and the 3′-Nucleotidase CpdB

**DOI:** 10.3390/molecules26082307

**Published:** 2021-04-16

**Authors:** Alicia Cabezas, Iralis López-Villamizar, María Jesús Costas, José Carlos Cameselle, João Meireles Ribeiro

**Affiliations:** 1 Grupo de Enzimología, Departamento de Bioquímica y Biología Molecular y Genética, Facultad de Medicina y Ciencias de la Salud, Universidad de Extremadura, 06006 Badajoz, Spain; acabezas@unex.es (A.C.); iralis80@hotmail.com (I.L.-V.); macostas@unex.es (M.J.C.); camselle@unex.es (J.C.C.); 2 Manlab, Diagnóstico Bioquímico y Genómico, Calle Marcelo Torcuato de Alvear 2263, 1122 Ciudad de Buenos Aires, Argentina

**Keywords:** chimeragenesis, protein domain, substrate-binding site, catalytic site, substrate specificity, 5′-nucleotidase, 3′-nucleotidase, phosphodiesterase, phosphoanhydride hydrolase

## Abstract

The 5′-nucleotidase UshA and the 3′-nucleotidase CpdB from *Escherichia coli* are broad-specificity phosphohydrolases with similar two-domain structures. Their N-terminal domains (UshA_Ndom and CpdB_Ndom) contain the catalytic site, and their C-terminal domains (UshA_Cdom and CpdB_Cdom) contain a substrate-binding site responsible for specificity. Both enzymes show only partial overlap in their substrate specificities. So, it was decided to investigate the catalytic behavior of chimeras bearing the UshA catalytic domain and the CpdB specificity domain, or vice versa. UshA_Ndom–CpdB_Cdom and CpdB_Ndom–UshA_Cdom were constructed and tested on substrates specific to UshA (5′-AMP, CDP-choline, UDP-glucose) or to CpdB (3′-AMP), as well as on 2′,3′-cAMP and on the common phosphodiester substrate bis-4-NPP (bis-4-nitrophenylphosphate). The chimeras did show neither 5′-nucleotidase nor 3′-nucleotidase activity. When compared to UshA, UshA_Ndom–CpdB_Cdom conserved high activity on bis-4-NPP, some on CDP-choline and UDP-glucose, and displayed activity on 2′,3′-cAMP. When compared to CpdB, CpdB_Ndom–UshA_Cdom conserved phosphodiesterase activities on 2′,3′-cAMP and bis-4-NPP, and gained activity on the phosphoanhydride CDP-choline. Therefore, the non-nucleotidase activities of UshA and CpdB are not fully dependent on the interplay between domains. The specificity domains may confer the chimeras some of the phosphodiester or phosphoanhydride selectivity displayed when associated with their native partners. Contrarily, the nucleotidase activity of UshA and CpdB depends strictly on the interplay between their native catalytic and specificity domains.

## 1. Introduction

Chimeragenesis is one of the tools of protein engineering applicable to the generation of novel enzyme specificities and to investigate the role of protein domains in biocatalysis [1,2]. This communication is a report of a first attempt to apply chimeragenesis to decipher the differential substrate specificity of two structurally related phosphohydrolases that have separate catalytic and specificity domains: the 5′-nucleotidase UshA and the 3′-nucleotidase CpdB from *Escherichia coli*.

UshA and CpdB are periplasmic enzymes. They are synthesized with a signal sequence that is removed upon protein export from the cytoplasm to the periplasm [3,4,5,6,7]. These enzymes, and their homologues in other gram-negative and gram-positive bacteria (in this case, they are cell wall-bound proteins), all act in the extracytoplasmic space, where they are involved in the recovery of extracellular nucleotide substrates and in the economy of phosphate [4,8,9,10,11,12,13]. The interest of their study is being enhanced by abundant evidence that they are modulators of bacterial virulence [14,15,16,17,18,19,20,21], interfere with the innate immune response of infected hosts, and are considered to be potential therapeutic targets in infectious diseases [20,22,23,24,25]. For this reason, deciphering the structural factors that determine their specificity is a priority.

**Figure 1:**
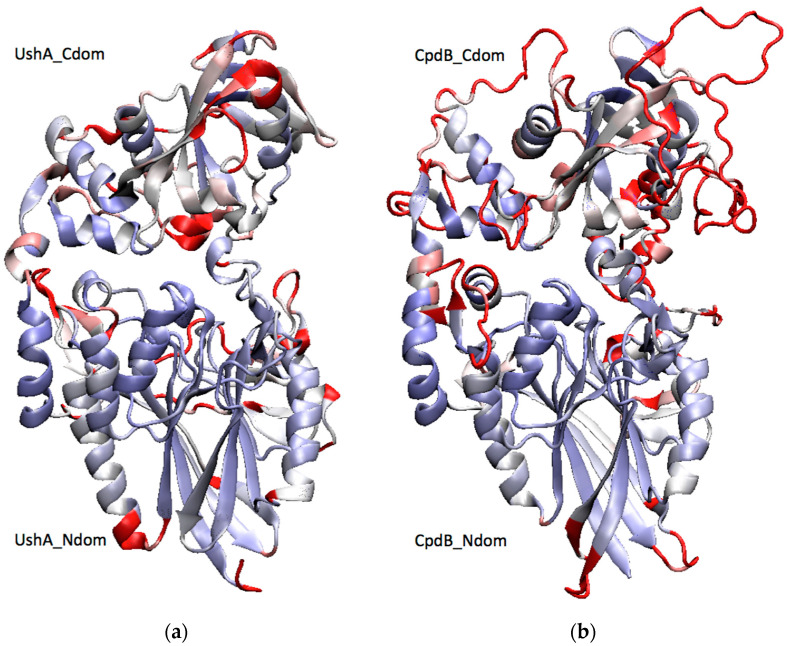
Two-domain structures of (**a**) UshA and (**b**) CpdB. A structural alignment of the crystal structure of a closed conformer of UshA (PDB: 1HPU, chain C) [30] with a homology model of CpdB [27] was generated with the VMD MultiSeq plugin [31], and it was used to color both proteins by structure conservation (blue, conserved; red, not conserved). UshA_Ndom, UshA_Cdom, CpdB_Ndom, and CpdB_Cdom (N-terminal and C-terminal domains of UshA or CpdB).

The structures of the 5′-nucleotidase UshA and the 3′-nucleotidase CpdB display the same two-domain architecture (Figure 1). They have an N-terminal metallophos domain (Pfam ID PF00149) that contains the catalytic site, including a dimetal center and a catalytic histidine (UshA_Ndom and CpdB_Ndom), and a C-terminal 5_nucleotid_C domain (Pfam ID PF02872) that contains a substrate-binding site, or specificity site, with two aromatic residues (UshA_Cdom and CpdB_Cdom) [26,27]. The N- and the C domains are joined together by a ≈20-amino acid linker [27,28]. In the case of UshA, the nucleotide substrate, e.g., 5′-AMP, binds to the specificity site in UshA_Cdom, with the adenine ring forming a stacking sandwich between two phenylalanine residues. Thereafter, UshA_Cdom undergoes a large hinge-bending rotation and it brings the substrate to the catalytic site in UshA_Ndom where hydrolysis takes place [29]. A similar scheme has been proposed for CpdB during 3′-nucleotidase catalysis [27]: 3′-AMP would bind to CpdB_Cdom, in this case with the adenine ring stacked between two tyrosine residues, followed by a rotation bringing the substrate to the catalytic site in CpdB_Ndom.

The enzyme specificities of UshA and CpdB have been defined by testing large lists of substrates, which depicted them as broad specificity, but still rather selective phosphohydrolases. UshA is a highly efficient 5′-nucleotidase, which is also very active on the phosphoanhydride linkages of CDP-alcohols and UDP-sugars, with catalytic efficiencies (*k*_cat_/*K*_M_) of 10^7^–10^8^ M^−1^s^−1^, being indicative of near diffusion-controlled rates [32,33]. It also shows high activity on bis-4-NPP (bis-4-nitrophenylphosphate) with Mn^2+^, Co^2+^ or Co^2+^/Ca^2+^ as activating cations [32,34], but not with Mg^2+^ [33]. It does not hydrolyze 2′,3′-cAMP in the presence of Mg^2+^ [33] or Co^2+^ and Ca^2+^ [34], but its possible Mn^2+^-dependent activity has not been studied [32]. CpdB is also a highly efficient 3′-nucleotidase and a phosphodiesterase of 2′,3′-cyclic mononucleotides and bis-4-NPP, with catalytic efficiencies of 10^6^–10^7^ M^−1^s^−1^ [35]. Remarkably, there is only partial overlap between the specificities of UshA and CpdB, as each one is not or very little active on most of the major substrates of the other, with the exception of bis-4-NPP, which is a very good substrate for both enzymes. Such a differential specificity, considering the very similar structures of both enzymes (Figure 1), prompted the question of what would be the catalytic behavior of enzyme chimeras bearing the catalytic domain of UshA and the specificity domain of CpdB, and vice versa. Therefore, UshA_Ndom–CpdB_Cdom and CpdB_Ndom–UshA_Cdom chimeras were constructed, and their activities assayed on a set of major substrates of either native UshA or native CpdB, to test whether the chimeras could display the selectivity of their specificity domains. The results partly confirmed this hypothesis, and allowed for reaching conclusions regarding the relevance of the interplay between the catalytic and specificity domains of UshA and CpdB in their native states, depending on the substrates participating in the reaction.

## 2. Results

### 2.1. Construction of the Chimeras and Confirmation of Their Molecular Identities

Plasmids pGEX-6P-3-UshA_Ndom–CpdB_Cdom and pGEX-6P-3-CpdB_Ndom–UshA_Cdom were constructed, as described under Section 4.1, by a combination of PCR amplifications, DNA ligations, and subcloning into two different vectors.

**Figure 2:**
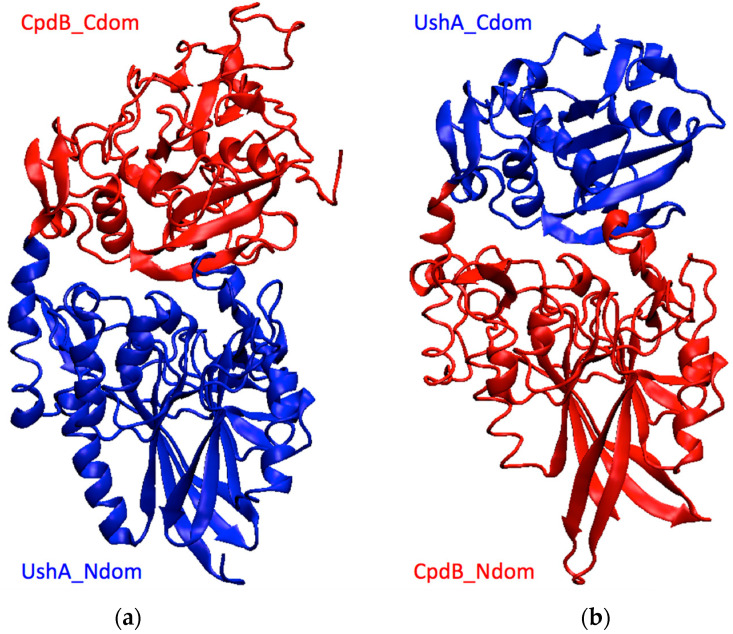
Models of the chimeras obtained by homology. (**a**) UshA_Ndom–CpdB_Cdom, (**b**) CpdB_Ndom–UshA_Cdom. Blue, UshA domains; red, CpdB domains.

The chimera coding sequences were confirmed by double-strand Sanger sequencing of plasmid passengers (Figures S1 and S2), and they were deposited in GenBank with accession numbers KP997254 (UshA_Ndom–CpdB_Cdom) and KP981373 (CpdB_Ndom–UshA_Cdom). These sequences were used to obtain homology models of both proteins (Section 4.2), which confirmed their theoretical ability to fold into two-domain structures that are similar to native UshA and CpdB (Figure 2).

The expression and purification of recombinant proteins was performed, as described under Section 4.3 from the above plasmids. The lysates of transformed BL21 cells showed protein bands that could correspond to glutathione S-tranferase (GST) fusions GST-UshA_Ndom–CpdB_Cdom (≈96.8 kDa) or to GST-CpdB_Ndom–UshA_Cdom (≈83.7 kDa) (Figure S3). After the removal of the GST tag, the recombinant proteins UshA_Ndom–CpdB_Cdom and CpdB_Ndom–UshA_Cdom bearing GPLGS extensions in the N term showed sizes that were in agreement with predictions from sequence (respectively, ≈70.4 kDa and ≈57.3 kDa) (Figure S3).

Further confirmation of the identities of GPLGS-UshA_Ndom–CpdB_Cdom and GPLGS-CpdB_Ndom–UshA_Cdom was sought by mass spectrometry. Tryptic peptide mass fingerprints were obtained from the 70.4 kDa and the 57.3 kDa bands, and they were compared to theoretical fingerprints derived from chimera protein sequences. The experimental fingerprints covered 87% of the sequence of GPLGS-UshA_Ndom–CpdB_Cdom (Figure S4 and Appendix S1) and 74% of GPLGS-CpdB_Ndom–UshA_Cdom (Figure S5 and Appendix S2). In both cases, the recorded mass signals included peptides of the N-terminal and C-terminal domains, and one of them included the GPLGS N-terminal extension. In the case of GPLGS-UshA_Ndom–CpdB_Cdom, one mass signal corresponded to a peptide that crosses the border between the two domains.

### 2.2. Substrate Specificity of the Chimeras

**Figure 3:**
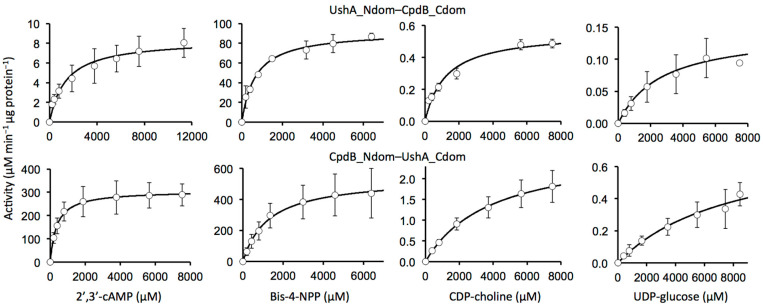
Saturation kinetics of chimeras with different substrates. The data points are shown as means ± standard deviations of the values that were obtained in three independent experiments.

A set of six substrates was selected to characterize the enzymatic behavior of the chimeras, including substrates specific to UshA (5′-AMP, CDP-choline, UDP-glucose) or to CpdB (3′-AMP), as well as 2′,3′-cAMP and the common phosphodiester substrate bis-4-NPP [32,33,34,35]. With these substrates, chimera activity assays were first run at a fixed, 750 µM concentration (Table 1). Measurable rates were obtained with all of them, except 5′-AMP and 3′-AMP, which gave rates that were below the detection limit with both chimeras. With the four substrates that gave measurable rates, saturation kinetics assays were run (Figure 3) and kinetic parameters *k*_cat_, *K*_M_ and *k*_cat_/*K*_M_ were obtained (Table 1).

The three major substrates specific to UshA either were not hydrolyzed by the chimera UshA_Ndom–CpdB_Ndom (5′-AMP) or were hydrolyzed at a low rate and catalytic efficiency (CDP-choline and UDP-glucose). With CpdB_Ndom–UshA_Ndom, 5′-AMP was not hydrolyzed, whereas CDP-choline and UDP-glucose were hydrolyzed at 5-fold and 1.6-fold increased rates with respect to native CpdB with a 20-fold and 10-fold increased *k*_cat_ values (Table 1).

The three major CpdB substrates tested (3′-AMP, 2′,3′-cAMP, bis-4-NPP) behaved differently to each other. First of all, 3′-AMP was not hydrolyzed either by the chimera UshA_Ndom–CpdB_Ndom or by CpdB_Ndom–UshA_Ndom. Things were different with 2′,3′-cAMP and bis-4-NPP. For one thing, as compared to native UshA, the chimera UshA_Ndom–CpdB_Ndom conserved a strong activity on bis-4-NPP and conserved (or perhaps gained) a modest one on 2′,3′-cAMP. For another, as compared to native CpdB, the chimera CpdB_Ndom–UshA_Ndom conserved much of the activity on these substrates, which were hydrolyzed at high rates with high catalytic efficiencies (Table 1).

## 3. Discussion

### 3.1. Limitations of the Study

In the construction of chimeras involving protein domains from different proteins, a factor that may affect the behavior of the recombined proteins is the length and flexibility of the linker used [36,37,38]. In this work, a direct path to domain recombination was followed, e.g., without testing different linker lengths, and taking advantage of the natural linkers of UshA and CpdB. They are proteins of moderate sequence homology, but very similar structure, and they are formed by two domains that are linked to each other by a natural, 20-amino acid spacer. UshA has been structurally characterized in detail [26,28,29,30,39,40,41,42], and the interdomain linker has been demonstrated to be flexible enough to allow a large hinge-bending rotation of its substrate-binding domain (UshA_Cdom) during the catalytic cycle [29]. CpdB has not been crystallographically studied, and its current model was derived by homology (anyhow, it should be remarked that CpdB modeling was independent on the structure of UshA) [27]. Accordingly, the flexibility of the CpdB linker, allowing a hinge-bending rotation of CpdB_Cdom, can be only inferred from the different established functions of its domains CpdB_Ndom and CpdB_Cdom and by analogy to UshA [27].

Concerning substrate specificity, UshA and CpdB are broad-specificity phosphohydrolases with only partial overlap in their substrate ranges. This study was limited to three major substrates of each enzyme, which, in part, delimit their specificities well and give opportunity to appraise the potential of chimeragenesis in its application to these enzymes. However, upon doing so, many other substrates were omitted, which are either relatively minor or, in a few cases, are hydrolyzed both by UshA and CpdB [33,35]. In addition, the substrate specificity of chimeras was only examined with Mn^2+^ as the activating cation. This coincides with the previous study of native CpdB [35], but as far as native UshA is concerned no similar control study is available, as published specificity reports rely on different metal-ion activators (Mg^2+^ [33], Mn^2+^ [32], Co^2+^, and Ca^2+^ [34]), and the published report on Mn^2+^-dependent activity does not include assays with 2′,3′-cAMP as substrate [32].

In the assays of chimera activities, relatively large standard deviations were obtained on several occasions. However, this does not affect the conclusions of the work.

### 3.2. Significance of the Study and Conclusions

To better follow this discussion, one should recall that the UshA_Ndom and CpdB_Ndom domains contain each the catalytic site of the corresponding native enzyme, and that UshA_Cdom and CpdB_Cdom each contain the substrate-binding site determinant of specificity.

The major aim of this study was to apply chimeragenesis, one of the methods of synthetic enzymology, to advance in the deciphering of UshA and CpdB specificities. In this respect, perhaps the major conclusion comes from a negative result: the absence of 5′-nucleotidase or 3′-nucleotidase activities in the chimeras constructed. UshA_Ndom–CpdB_Cdom and CpdB_Ndom–UshA_Cdom both failed to show the natural nucleotidase activity of their catalytic subunits, as much as the presence of the alien specificity domains failed to induce their chimeric catalytic partners to change their nucleotidase specificity. This indicates that the presence of the correct domain pair is essential for the nucleotidase activities, and it supports that the specificities of the native enzymes for either 5′-nucleotides or 3′-nucleotides depend on a delicate interplay between the catalytic and specificity domains.

The activities of the chimeras on phosphoanhydride and phosphodiester substrates can be considered from two different points of view.

On the one hand, one can consider the activities of each chimera on the phosphoanhydride or phosphodiester substrates proper of the native enzyme (UshA or CpdB) that contributes with the catalytic domain (UshA_Ndom–CpdB_Cdom or CpdB_Ndom–UshA_Cdom, respectively). This point of view revealed that the native domain pair is not essential for these activities. This conclusion is strongly supported for CpdB, because CpdB_Ndom–UshA_Cdom conserved very high phosphodiesterase activities on 2′,3′-cAMP and bis-4-NPP, despite the absence of the specificity domain of CpdB. Regarding UshA activities on phosphoanhydrides, the non-essentiality of the correct domain pair is less marked, because the activities of UshA_Ndom–CpdB_Cdom on CDP-choline and UDP-glucose was significant, but very low.

From a different point of view, one can consider the activities of the chimeras on the substrates proper of the native enzyme that contributes with the specificity domain. This point of view revealed that, in the chimeras, alien specificity-domains somewhat influenced the catalytic sites. This is clearly so in the case of CpdB_Ndom–UshA_Cdom, since, when compared to native CpdB, it showed a gain of activity on phosphoanhydrides CDP-choline and UDP-glucose, particularly so in terms of *k*_cat_. This could be also true for UshA_Ndom–CpdB_Cdom, which maintained significant levels of activity on the phosphodiesters bis-4-NPP and 2′,3′-cAMP, although this could just be a consequence of activity conservation with respect to native UshA.

In summary, the study of these chimeric enzymes sheds new light to decipher the molecular bases of UshA and CpdB specificities, through the six following conclusions: (i) the nucleotidase activities of UshA and CpdB strictly depend on the presence of the native combination of catalytic and specificity domains, possibly through a delicate interplay between them; (ii) the UshA activities on phosphoanhydride substrates are somewhat tolerant to the substitution of the native specificity domain by that of CpdB; (iii) the CpdB activities on phosphodiester substrates are largely tolerant to the substitution of the native specificity domain by that of UshA; (iv) the phosphodiesterase activities of native UshA are partly conserved (or perhaps increased in the case of 2′,3′-cAMP) by the presence of the CpdB specificity domain in the corresponding chimeric enzyme; (v) the lack of CpdB activity on phosphoanhydride substrates is increased by the presence of the UshA specificity domain in the corresponding chimeric enzyme; and, (vi) the non-nucleotidase activities of UshA and CpdB are not strictly dependent on the native combination of catalytic and specificity domains.

## 4. Materials and Methods

### 4.1. Construction of the Chimeras

**Figure 4:**
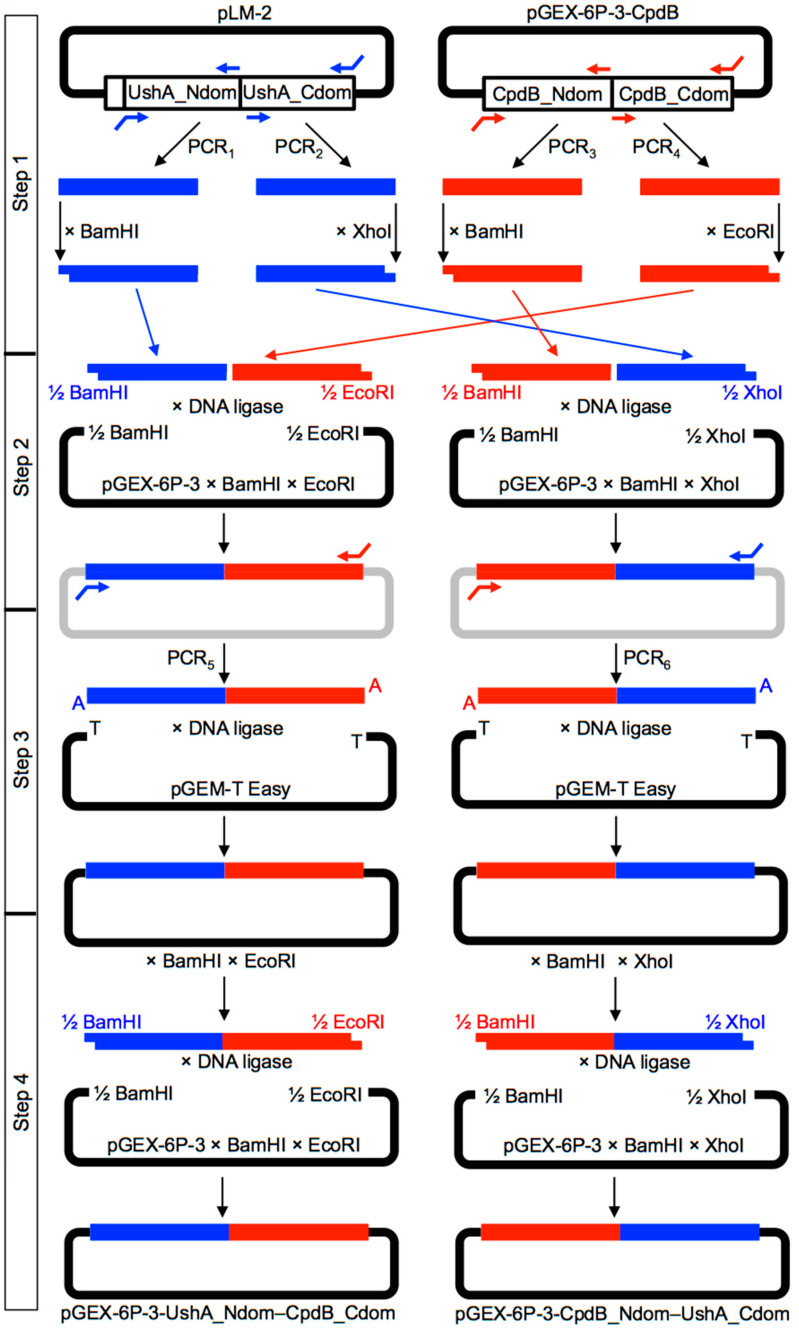
Strategy followed to construct DNA sequences encoding UshA_Ndom–CpdB_Cdom and CpdB_Ndom–UshA_Cdom chimeras. Table S1 shows the primers used for PCR_1_–PCR_6_.

UshA_Ndom–CpdB_Cdom and CpdB_Ndom–UshA_Cdom coding sequences were constructed in four steps, as summarized in Figure 4.

Step 1. The sequences encoding the four protein domains needed were separately amplified by PCR from plasmids pLM-2 (PCR_1_–PCR_2_), containing the coding sequence of the precursor of mature UshA [43], and from pGEX-6P3-cpdB (PCR_3_–PCR_4_), containing the coding sequence of mature CpdB [35]. For UshA domains, the primers were designed to incorporate BamHI and XhoI sites at the start of UshA_Ndom (PCR_1_) and at the end of UshA_Cdom (PCR_2_), while leaving blunt, non-cuttable ends at the end of UshA_Ndom and at the start of UshA_Cdom. For CpdB domains, the primers were designed to incorporate BamHI and EcoRI sites at the start of CpdB_Ndom (PCR_3_) and at the end of CpdB_Cdom (PCR_4_), while leaving blunt, non-cuttable ends at the end of CpdB_Ndom and at the start of CpdB_Cdom. The four amplicons obtained with *PfuTurbo* DNA polymerase (Agilent Technologies Spain S.L., Las Rozas de Madrid, Spain) were separately digested with the enzyme recognizing the corresponding site added by PCR.

Step 2. Two triple ligations were implemented with T4 DNA ligase (New England Biolabs; purchased from C. Viral S.L., Sevilla, Spain). One mixture included the BamHI-digested UshA_Ndom amplicon, the EcoRI-digested CpdB_Cdom amplicon, and the pGEX-6P-3 plasmid digested with BamHI and EcoRI. Another mixture included the BamHI-digested CpdB_Ndom amplicon, the XhoI-digested UshA_Cdom amplicon, and the pGEX-6P-3 plasmid digested with BamHI and XhoI. These triple ligation reactions were judged to be difficult, because each involved one blunt-end ligation and two cohesive-end ligations, and very low yields of the desired products were expected.

Step 3. In order to overcome the difficulties of step 2, both ligation mixtures were used as templates for an additional PCR using, in each case, the primer pair expected to hybridize with the ends of the chimeric DNAs encoding UshA_Ndom–CpdB_Cdom or CpdB_Ndom–UshA_Cdom, respectively (PCR_5_ and PCR_6_). In these cases, a DNA polymerase mix that leaves 3′-A overhangs was used (Advantage cDNA Polymerase Mix, Clontech, available from Takara Bio Europe SAS, Saint-Germain-en-Laye, France). The amplicons obtained were ligated with the linearized vector pGEM-T Easy bearing 3′-T overhangs (pGEM-T Easy Vector System I, Promega Biotech Ibérica S.L., Alcobendas, Spain).

Step 4. The pGEM-T Easy constructions encoding UshA_Ndom–CpdB_Cdom or CpdB_Ndom–UshA_Cdom were digested with BamHI and EcoRI or with BamHI and XhoI, respectively, to obtain DNA fragments that were ready to be subcloned in pGEX-6P-3 digested with the same enzymes, which gave plasmids pGEX-6P-3-UshA_Ndom–CpdB_Cdom and pGEX-6P-3-CpdB_Ndom–UshA_Cdom. The sequence of the chimeric plasmids was confirmed by double-strand Sanger sequencing (Figures S1 and S2) (Servicio de Genómica, Instituto de Investigaciones Biomédicas “Alberto Sols”, Consejo Superior de Investigaciones Científicas, Universidad Autónoma, Madrid).

### 4.2. Homology Modeling of Chimeric Proteins

Homology modeling of the chimeric proteins was performed in the Phyre2 server (http://www.sbg.bio.ic.ac.uk/phyre2/, accessed on 24 March 2021) [44]. In the case of UshA_Ndom–CpdB_Cdom (size, 692 residues), a run in ‘intensive mode’ was needed to obtain a model that covered the full length of the chimera. The structure PDB ID 1oid was automatically selected as a template, although 109 residues were modeled by ab initio. In the case of CpdB_Ndom–UshA_Cdom (size, 522 residues), a standard run in ‘normal mode’ returned a model that covered chimera residues 2–522, again using 1oid as template.

### 4.3. Expression and Purification of Chimeric Proteins

BL-21 *E. coli* colonies bearing either pGEX-6P-3-UshA_Ndom–CpdB_Cdom or pGEX-6P-3-CpdB_Ndom–UshA_Cdom, and, therefore, resistant to ampicillin, were amplified in culture and were induced with isopropylthiogalactoside (IPTG) to activate transcription from the *tac* promoter present in the pGEX-6P-3 vector. The GST fusion proteins, GST-UshA_Ndom–CpdB_Cdom and GST-CpdB_Ndom–UshA_Cdom, were recovered in the supernatant of bacterial lysates (Figure S3) and they were applied to GSH-Sepharose columns. In this step, the GST fusions were adsorbed by affinity. Thereafter, the chimeric enzymes that were bound to the affinity gel were separated from the GST tag, and henceforth released from the gel, by overnight incubation with Prescission protease (GE Healthcare; purchased from VWR International Eurolab SLU, Llinars del Vallés, Spain). This left an N-terminal GPLGS extension in the chimeric proteins (Figures S1 and S2). The final purity achieved (62% for GPLGS-UshA_Ndom–CpdB_Cdom and 85% for GST-CpdB_Ndom–UshA_Cdom) was estimated by sodium-dodecylsulfate polyacrylamide gel electrophoresis (SDS-PAGE, Figure S3) that was stained with Coomassie Blue (see below) and quantitated with GelAnalyzer 2010 (http://www.gelanalyzer.com, accessed on 29 November 2020). The protein content was assayed according to Bradford [45].

SDS-PAGE staining was performed by the incubation of the gel for 60 min. in a solution of Coomassie Blue (1 g L^−1^) in 50% methanol (by vol.) and 10% acetic acid (by vol.), followed by a several-hour wash with 10% methanol (by vol.) and 10% acetic acid (by vol.) until the satisfactory removal of the background. Whenever the protein bands were to be used for peptide mass fingerprinting (see below), the staining time was shortened to 20 min. and the several-hour wash was performed with 10% methanol (by vol.).

### 4.4. Tryptic Peptide Mass Fingerprinting

The peptide mass fingerprints of the chimeric proteins were obtained from protein bands that were cut out of SDS-PAGE gels, which had been stained with Coomassie Blue by the special protocol described above. The protein bands were sent to be processed and analyzed in the Unidad de Proteómica del Centro de Genómica y Proteómica, Facultad de Farmacia, Universidad Complutense de Madrid. The proteins were extracted from the gel pieces, digested with trypsin, and then analyzed by Matrix-Assisted Laser Desorption Ionization Time-Of-Flight (MALDI-TOF) mass spectrometry in a 4700 Proteomic Analyzer (Applied Biosystems). The list of masses of the recorded signals were compared to the theoretical tryptic-peptide masses that were predicted with the program PeptideMap (http://prowl.rockefeller.edu/prowl/peptidemap.html, accessed on 30 July 2015, currently discontinued) for the amino acid sequence of the corresponding chimeric protein. For these comparisons, cysteine carbamidomethylation was considered as a fixed modification, methione oxidation was considered as a variable one, up to three missed trypsin cuts were allowed, and the masses were computed as coincident within a 100 ppm error. The full reports that were downloaded from PeptideMap are included in the Supplementary Material as Appendix S1 and Appendix S2.

### 4.5. Activity Assays and Determination of Kinetic Parameters

The Mn^2+^-dependent activity assays were performed, as described [35]. In brief, the liberation of inorganic phosphate from all of the substrates was quantitated by a sensitive colorimetric assay. The nucleotidase activities on 5′-AMP or 3′-AMP were estimated from the direct liberation of phosphate by the chimeric enzymes. The phosphodiesterase activities on 2′,3′-cAMP or bis-4-NPP, and the phosphoanhydride hydrolase activities on CDP-choline or UDP-glucose, were assayed in the presence of alkaline phosphatase as auxiliary enzyme, taking advantage of the fact that these substrates are resistant to phosphatase, but the products that are formed by the chimeric enzymes are not. To determine the kinetic parameters *k*_cat_, *K*_M_ and *k*_cat_/*K*_M_, enzyme saturation experiments were run at varying substrate concentrations and the Michaelis–Menten equation was adjusted to the data points by nonlinear regression using the Solver function of Microsoft Excel.

## Figures and Tables

**Table 1:** Substrate specificity and kinetic parameters of UshA_Ndom–CpdB_Cdom and CpdB_Ndom–UshA_Cdom. All of the data for chimeras were obtained with Mn^2+^ as the activating cation. The kinetic parameters were derived from the saturation curves that are shown in Figure 3. The results are expressed as mean values ± standard deviations of 3–8 replicates. Native-enzyme data are from earlier work (UshA [32,33,34]; CpdB [35]) shown here only for comparison with the chimeras: all of them correspond to Mn^2+^-dependent activities [32,35], except some UshA data that were obtained with Mg^2+^ [33] or Co^2+^ and Ca^2+^ [34].

Protein	Substrate	Rate at Fixed Substrate Concentration (750 µM) ^1^	*k*_cat_	*K*_M_	*k*_cat_ */K*_M_
		nmol min^−1^ mg^−1^	s^−1^	µM	M^−1^ s^−1^
UshA_Ndom–CpdB_Cdom	5′-AMP	<3	na	na	na
	CDP-choline	83 ± 8	0.3 ± 0.01	1400 ± 400	210 ± 60
	UDP-glucose	12 ± 4	0.1 ± 0.01	5000 ± 1700	20 ± 10
	3′-AMP	<3	na	na	na
	2′,3′-cAMP	1900 ± 1060	3.6 ± 1.0	1100 ± 350	3400 ± 1300
	Bis-4-NPP	20,500 ± 2700	45 ± 3	670 ± 120	68,000 ± 13,000
CpdB_Ndom–UshA_Cdom	5′-AMP	<3	na	na	na
	CDP-choline	1800 ± 210	11 ± 3	3900 ± 600	2700 ± 300
	UDP-glucose	27 ± 6	0.2 ± 0.1	5900 ± 1900	40 ± 8
	3′-AMP	<3	na	na	na
	2′,3′-cAMP	75,000 ± 15,000	117 ± 24	370 ± 40	320,000 ± 80,000
	Bis-4-NPP	71,000 ± 18,000	208 ± 77	1400 ± 60	150,000 ± 55,000
Native UshA [32] (or as referenced in parenthesis)	5′-AMP	360,000 (135,000 [33])	ns (372 [33])	ns (1.8 [33])	ns (2 × 10^8^ [33])
	CDP-choline	ns (51,000 [33])	ns (231 [33])	ns (2.4 [33])	ns (10^8^ [33])
	UDP-glucose	131,000 (16,000 [33])	504 (71 [33])	45 (10 [33])	10^7^ (10^7^ [33])
	3′-AMP	0 (0 [34])	na	na	na
	2′,3′-cAMP	ns (800 [33]; 0 [34])	ns	ns	ns
	Bis-4-NPP	537,000 (800 [33])	ns	ns	ns
Native CpdB [35]	5′-AMP	4	na	na	na
	CDP-choline	370	0.5	219	2300
	UDP-glucose	17	0.02	392	28
	3′-AMP	147,000	176	14	1.3 × 10^7^
	2′,3′-cAMP	123,000	190	27	7.3 × 10^6^
	Bis-4-NPP	127,000	340	96	3.6 × 10^6^

^1^ 250 µM in the case of native UshA [32]. Bis-4-NPP, bis-4-nitrophenylphosphate; na, not assayable (activity below detection threshold); ns, not studied.

## Data Availability

The data presented in this study are available in the article and its supplementary material.

## Supplementary Materials

The following are available online, Table S1: PCR primers used to construct plasmids pGEX-6P-3-UshA_Ndom–CpdB_Cdom and pGEX-6P-3-CpdB_Ndom–UshA_Cdom, Figure S1: Sanger sequencing of the pGEX-6P-3-UshA_Ndom–CpdB_Cdom plasmid, Figure S2: Sanger sequencing of the pGEX-6P-3-CpdB_Ndom–UshA_Cdom plasmid, Figure S3: Expression of the recombinant proteins from plasmids pGEX-6P-3-UshA_Ndom–CpdB_Cdom and pGEX-6P-3-CpdB_Ndom–UshA_Cdom, Figure S4: Identification of the recombinant protein GPLGS-UshA_Ndom–CpdB_Cdom by its peptide mass fingerprint, Figure S5: Identification of the recombinant protein GPLGS-CpdB_Ndom–UshA_Cdom by its peptide mass fingerprint, Appendix S1: PeptideMap report on the peptide mass fingerprint of the recombinant protein GPLGS-UshA_Ndom–CpdB_Cdom, Appendix S2: PeptideMap report on the peptide mass fingerprint of the recombinant protein GPLGS-CpdB_Ndom–UshA_Cdom.

## Sample Availability

Samples of the plasmids encoding the chimeras are available from the authors upon request.

## Abbreviations

Bis-4-NPP	Bis-4-nitrophenylphosphate
CpdB_Cdom	C-terminal domain of CpdB
CpdB_Ndom	N-terminal domain of CpdB
GST	Glutathione S-transferase
SDS-PAGE	Sodium dodecyl sulfate polyacrylamide gel electrophoresis
UshA_Cdom	C-terminal domain of UshA
UshA_Ndom	N-terminal domain of UshA

## References

[1] Goodey N.M., Benkovic S.J., Köhrer G., RajBhandary U.L. (2009). Understanding enzyme mechanism through protein chimeragenesis. Protein Engineering.

[2] Dinis P., Wandi B.N., Grocholski T., Metsä-Ketelä M., Singh R.S., Singhania R.R., Pandey A., Larroche C. (2019). Chimeragenesis for biocatalysis. Advances in Enzyme Technology.

[3] Cowman A., Beacham I.R. (1980). Molecular cloning of the gene (*ush*) from *Escherichia coli* specifying periplasmic UDP-sugar hydrolase (5′-nucleotidase). Gene.

[4] Beacham I.R., Garrett S. (1980). Isolation of *Escherichia coli* mutants (*cpdB*) deficient in periplasmic 2′:3′-cyclic phosphodiesterase and genetic mapping of the *cpdB* locus. J. Gen. Microbiol..

[5] Burns D.M., Abraham L.J., Beacham I.R. (1983). Characterization of the ush gene of *Escherichia coli* and its protein products. Gene.

[6] Burns D.M., Beacham I.R. (1986). Nucleotide sequence and transcriptional analysis of the *E. coli ushA* gene, encoding periplasmic UDP-sugar hydrolase (5′-nucleotidase): Regulation of the *ushA* gene, and the signal sequence of its encoded protein product. Nucleic Acids Res..

[7] Liu J., Burns D.M., Beacham I.R. (1986). Isolation and sequence analysis of the gene (*cpdB*) encoding periplasmic 2′,3′-cyclic phosphodiesterase. J. Bacteriol..

[8] Beacham I.R., Kahana R., Levy L., Yagil E. (1973). Mutants of *Escherichia coli* K-12 ”cryptic,” or deficient in 5′-nucleotidase (uridine diphosphate-sugar hydrolase) and 3′-nucleotidase (cyclic phosphodiesterase) activity. J. Bacteriol..

[9] Yagil E., Beacham I.R. (1975). Uptake of adenosine 5′-monophosphate by *Escherichia coli*. J. Bacteriol..

[10] Trülzsch K., Roggenkamp A., Pelludat C., Rakin A., Jacobi C., Heesemann J. (2001). Cloning and characterization of the gene encoding periplasmic 2′,3′-cyclic phosphodiesterase of *Yersinia enterocolitica* O:8. Microbiology.

[11] Rittmann D., Sorger-Herrmann U., Wendisch V.F. (2005). Phosphate starvation-inducible gene *ushA* encodes a 5′ nucleotidase required for growth of *Corynebacterium glutamicum* on media with nucleotides as the phosphorus source. Appl. Environ. Microbiol..

[12] Kakehi M., Usuda Y., Tabira Y., Sugimoto S. (2007). Complete deficiency of 5′-nucleotidase activity in *Escherichia coli* leads to loss of growth on purine nucleotides but not of their excretion. J. Mol. Microbiol. Biotechnol..

[13] McDonough E., Kamp H., Camilli A. (2016). *Vibrio cholerae* phosphatases required for the utilization of nucleotides and extracellular DNA as phosphate sources. Mol. Microbiol..

[14] Osaki M., Takamatsu D., Shimoji Y., Sekizaki T. (2002). Characterization of *Streptococcus suis* genes encoding proteins homologous to sortase of gram-positive bacteria. J. Bacteriol..

[15] Metcalf D.S., MacInnes J.I. (2007). Differential expression of *Haemophilus parasuis* genes in response to iron restriction and cerebrospinal fluid. Can. J. Vet. Res..

[16] Li W., Liu L., Chen H., Zhou R. (2009). Identification of *Streptococcus suis* genes preferentially expressed under iron starvation by selective capture of transcribed sequences. FEMS Microbiol. Lett..

[17] Tseng S.P., Lin Y.Y., Tsai J.C., Hsueh P.R., Chen H.J., Hung W.C., Teng L.J. (2010). Distribution of *emm* types and genetic characterization of the *mgc* locus in group G *Streptococcus dysgalactiae* subsp. equisimilis from a hospital in northern Taiwan. J. Clin. Microbiol..

[18] Liu H., Chen L., Wang X., Si W., Wang H., Wang C., Liu S., Li G. (2015). Decrease of colonization in the chicks′ cecum and internal organs of *Salmonella enterica* serovar Pullorum by deletion of *cpdB* by Red system. Microb. Pathog..

[19] Liu H., Chen L., Si W., Wang C., Zhu F., Li G., Liu S. (2017). Physiology and pathogenicity of *cpdB* deleted mutant of avian pathogenic *Escherichia Coli*. Res. Vet. Sci..

[20] Andrade W.A., Firon A., Schmidt T., Hornung V., Fitzgerald K.A., Kurt-Jones E.A., Trieu-Cuot P., Golenbock D.T., Kaminski P.A. (2016). Group B *Streptococcus* degrades cyclic-di-AMP to modulate STING-dependent type I interferon production. Cell Host Microbe.

[21] Deng S., Xu T., Fang Q., Yu L., Zhu J., Chen L., Liu J., Zhou R. (2018). The surface-exposed protein SntA contributes to complement evasion in zoonotic *Streptococcus Suis*. Front. Immunol..

[22] Dey R.J., Dey B., Zheng Y., Cheung L.S., Zhou J., Sayre D., Kumar P., Guo H., Lamichhane G., Sintim H.O. (2017). Inhibition of innate immune cytosolic surveillance by an *M. tuberculosis* phosphodiesterase. Nat. Chem. Biol..

[23] Li L. (2017). Host-Pathogen interactions: Nucleotide circles of life and death. Nat. Chem. Biol..

[24] Devaux L., Kaminski P.A., Trieu-Cuot P., Firon A. (2018). Cyclic di-AMP in host-pathogen interactions. Curr. Opin. Microbiol..

[25] Eaglesham J.B., Kranzusch P.J. (2020). Conserved strategies for pathogen evasion of cGAS-STING immunity. Curr. Opin. Immunol..

[26] Krug U., Patzschke R., Zebisch M., Balbach J., Sträter N. (2013). Contribution of the two domains of *E. coli* 5′-nucleotidase to substrate specificity and catalysis. FEBS Lett..

[27] López-Villamizar I., Cabezas A., Pinto R.M., Canales J., Ribeiro J.M., Rodrigues J.R., Costas M.J., Cameselle J.C. (2021). Molecular Dissection of *Escherichia* coli CpdB: Roles of the N Domain in Catalysis and Phosphate Inhibition, and of the C Domain in Substrate Specificity and Adenosine Inhibition. Int. J. Mol. Sci..

[28] Knöfel T., Sträter N. (1999). X-ray structure of the *Escherichia coli* periplasmic 5′-nucleotidase containing a dimetal catalytic site. Nat. Struct. Biol..

[29] Knöfel T., Sträter N.E. (2001). *coli* 5′-nucleotidase undergoes a hinge-bending domain rotation resembling a ball-and-socket motion. J. Mol. Biol..

[30] Knöfel T., Sträter N. (2001). Mechanism of hydrolysis of phosphate esters by the dimetal center of 5′-nucleotidase based on crystal structures. J. Mol. Biol..

[31] Roberts E., Eargle J., Wright D., Luthey-Schulten Z. (2006). MultiSeq: Unifying sequence and structure data for evolutionary analysis. BMC Bioinform..

[32] Ruiz A., Hurtado C., Ribeiro J.M., Sillero A., Sillero M.A.C. (1989). Hydrolysis of bis(5′-nucleosidyl) polyphosphates by *Escherichia coli* 5′-nucleotidase. J. Bacteriol..

[33] Alves-Pereira I., Canales J., Cabezas A., Cordero P.M., Costas M.J., Cameselle J.C. (2008). CDP-alcohol hydrolase, a very efficient activity of the 5′-nucleotidase/UDP-sugar hydrolase encoded by the *ushA* gene of *Yersinia intermedia* and *Escherichia coli*. J. Bacteriol..

[34] Neu H.C. (1967). The 5′-nucleotidase of *Escherichia coli*. I. Purification and properties. J. Biol. Chem..

[35] López-Villamizar I., Cabezas A., Pinto R.M., Canales J., Ribeiro J.M., Cameselle J.C., Costas M.J. (2016). The characterization of *Escherichia coli* CpdB as a recombinant protein reveals that, besides having the expected 3′-nucleotidase and 2′,3′-cyclic mononucleotide phosphodiesterase activities, it is also active as cyclic dinucleotide phosphodiesterase. PLoS ONE.

[36] Corbi-Verge C., Marinelli F., Zafra-Ruano A., Ruiz-Sanz J., Luque I., Faraldo-Gómez J.D. (2013). Two-state dynamics of the SH3-SH2 tandem of Abl kinase and the allosteric role of the N-cap. Proc. Natl. Acad. Sci. USA.

[37] Chen X., Zaro J.L., Shen W.C. (2013). Fusion protein linkers: Property, design and functionality. Adv. Drug Deliv. Rev..

[38] Huang Z., Zhang C., Xing X.H. (2021). Design and construction of chimeric linker library with controllable flexibilities for precision protein engineering. Methods Enzymol..

[39] Schultz-Heienbrok R., Maier T., Sträter N. (2004). Trapping a 96 degrees domain rotation in two distinct conformations by engineered disulfide bridges. Protein Sci..

[40] Schultz-Heienbrok R., Maier T., Sträter N. (2005). A large hinge bending domain rotation is necessary for the catalytic function of *Escherichia coli* 5′-nucleotidase. Biochemistry.

[41] Sträter N. (2006). Ecto-5′-nucleotidase: Structure function relationships. Purinergic Signal.

[42] Krug U., Alexander N.S., Stein R.A., Keim A., McHaourab H.S., Sträter N., Meiler J. (2015). Characterization of the domain orientations of *E. coli* 5′-nucleotidase by fitting an ensemble of conformers to DEER distance distributions. Structure.

[43] McMillen L., Beacham I.R., Burns D.M. (2003). Cobalt activation of *Escherichia coli* 5′-nucleotidase is due to zinc ion displacement at only one of two metal-ion-binding sites. Biochem. J..

[44] Kelley L.A., Mezulis S., Yates C.M., Wass M.N., Sternberg M.J. (2015). The Phyre2 web portal for protein modeling, prediction and analysis. Nat. Protoc..

[45] Bradford M.M. (1976). A rapid and sensitive method for the quantitation of microgram quantities of protein utilizing the principle of protein-dye binding. Anal. Biochem..

