# Supplementary material for: Substrate Specificity of Chimeric Enzymes Formed by Interchange of the Catalytic and Specificity Domains of the 5′-Nucleotidase UshA and the 3′-Nucleotidase CpdB
Source: Molecules. 2021 Apr 16;26(8):2307. doi: 10.3390/molecules26082307 (PMC8071527; doi:10.3390/molecules26082307)
Supplement: Supplementary file 1 [file molecules-26-02307-s001.zip › molecules-1156854-supplementary.pdf]

Supplementary Table, Figures and Appendixes to:

Substrate Specificity of Chimeric Enzymes Formed by Interchange of the Catalytic and Specificity Domains of the 5′-Nucleotidase UshA and the 3′-Nucleotidase CpdB

Alicia Cabezas, Iralis López-Villamizar, María Jesús Costas, José Carlos Cameselle and João Meireles Ribeiro,

This document contains the following supplementary Table, Figures and Appendixes:

Table S1. PCR primers used to construct plasmids pGEX-6P-3-UshA_Ndom–CpdB_Cdom and pGEX-6P-3-CpdB_Ndom–UshA_Cdom.

Figure S1. Sanger sequencing of the pGEX-6P-3-UshA_Ndom–CpdB_Cdom plasmid.

Figure S2. Sanger sequencing of the pGEX-6P-3-CpdB_Ndom–UshA_Cdom plasmid.

Figure S3. Expression of the recombinant proteins from plasmids pGEX-6P-3-UshA_Ndom–CpdB_Cdom and pGEX-6P-3-CpdB_Ndom–UshA_Cdom.

Figure S4. Identification of the recombinant protein GPLGS-UshA_Ndom–CpdB_Cdom by its peptide mass fingerprint.

Figure S5. Identification of the recombinant protein GPLGS-CpdB_Ndom–UshA_Cdom by its peptide mass fingerprint.

Appendix S1. PeptideMap report on the peptide mass fingerprint of the recombinant protein GPLGS-UshA_Ndom–CpdB_Cdom.

Appendix S2. PeptideMap report on the peptide mass fingerprint of the recombinant protein GPLGS-CpdB_Ndom–UshA_Cdom.

Table S1. PCR primers used to construct plasmids pGEX-6P-3-UshA_Ndom–CpdB_Cdom and pGEX-6P-3-CpdB_Ndom–UshA_Cdom.

1	UshA_Nterm-Fow	CACTGGGGATCCTATGAGCA***A***GATAAAACC
	Translation	Y E Q D K T
2	UshA_Nterm-Rev	CACTTCCAGCTGCGCTTTGCC
	Reverse complement	ggcaaagcgcagctggaagtg
	Translation	G K A Q L E V
3	UshA_Cterm-Fow	AAGATAGG***T***GAAACCAATGGTCGTCTGG
	Translation	K I G E T N G R L
4	UshA_Cterm-Rev	CATACGCTCGAGTTACTGCCAGCTCACCTCACC
	Reverse complement	ggtgaggtgagctggcagtaactcgagcgtatg
	Translation	G E V S W Q *
5	CpdB_Nterm-Fow	CACTGGGGATCCGCGAC***A***GTCGATCTACGTATCATGGAAACCACTG
	Translation	A T V D L R I M E T T
6	CpdB_Nterm-Rev_b	CTTGCTGACGAACTGGCGTGTGGC
	Reverse complement	gccacacgccagttcgtcagcaag
	Translation	A T R Q F V S K
7	CpdB_Cterm-Fow_b	CCAAT***T***GGTAAATCCGCCGACAATATG
	Translation	P I G K S A D N M
8	CpdB_Cterm-Rev	CTGCACGAATTCTTACTTACTCAAATCCACCTGATAAATCGCAAACCCG
	Reverse complement	cgggtttgcgatttatcaggtggatttgagtaagtaagaattcgtgcag
	Translation	G F A I Y Q V D L S K *

1. Forward primer used in combination with primer 2 (PCR_1, Figure 3) to amplify the coding sequence of the N domain of mature UshA (UshA_Ndom). It includes a BamHI site (underlined) before the coding sequence, and it bears a designed silent substitution (italics bod type) with respect to the pLM-2 passenger. It was also used in combination with primer 8 (PCR_5, Figure 3) to amplify the coding sequence of the UshA_Ndom–CpdB_Cdom chimera.
2. Reverse primer used in combination with primer 1 (PCR_1, Figure 3) to amplify the coding sequence of the N domain of mature UshA (UshA_Ndom). It bears no end additions or modifications of the coding sequence.
3. Forward primer used in combination with primer 4 (PCR_2, Figure 3) to amplify the coding sequence of the C domain of mature UshA (UshA_Cdom). It bears no end additions but contains a designed silent substitution (italics bod type) with respect to the pLM-2 passenger.
4. Reverse primer used in combination with primer 3 (PCR_2, Figure 3) to amplify the coding sequence of the C domain of mature UshA (UshA_Cdom). It includes an XhoI site (underlined) before the reverse complement of the coding sequence. It was also used in combination with primer 5 (PCR_6, Figure 3) to amplify the coding sequence of the CpdB_Ndom–UshA_Cdom chimera.
5. Forward primer used in combination with primer 6 (PCR_3, Figure 3) to amplifiy the coding sequence of the N domain of mature CpdB (CpdB_Ndom). It includes a BamHI site (underlined) before the coding sequence, and it bears a designed silent substitution with respect to GenBank accession KP938772 (italics bold type). It was also used in combination with primer 4 (PCR_6, Figure 3) to amplify the coding sequence of the CpdB_Ndom–UshA_Cdom chimera.
6. Reverse primer used in combination with primer 5 (PCR_3, Figure 3) to amplifiy the coding sequence of the N domain of mature CpdB (CpdB_Ndom). It bears no end additions or modifications of the coding sequence.
7. Forward primer used in combination with primer 8 (PCR_4, Figure 3) to amplify the coding sequence of the C domain of mature CpdB (CpdB_Cdom). It bears no end additions but contains a designed silent substitution (italics bod type) with respect to GenBank accession KP938772 (italics bold type).
8. Reverse primer used in combination with primer 7 (PCR_4, Figure 3) to amplify the coding sequence of the C domain of mature CpdB (CpdB_Cdom). It includes an EcoRI site (underlined) before the reverse complement of the coding sequence. It was also used in combination with primer 1 (PCR_5, Figure 3) to amplify the coding sequence of the UshA_Ndom–CpdB_Cdom chimera.

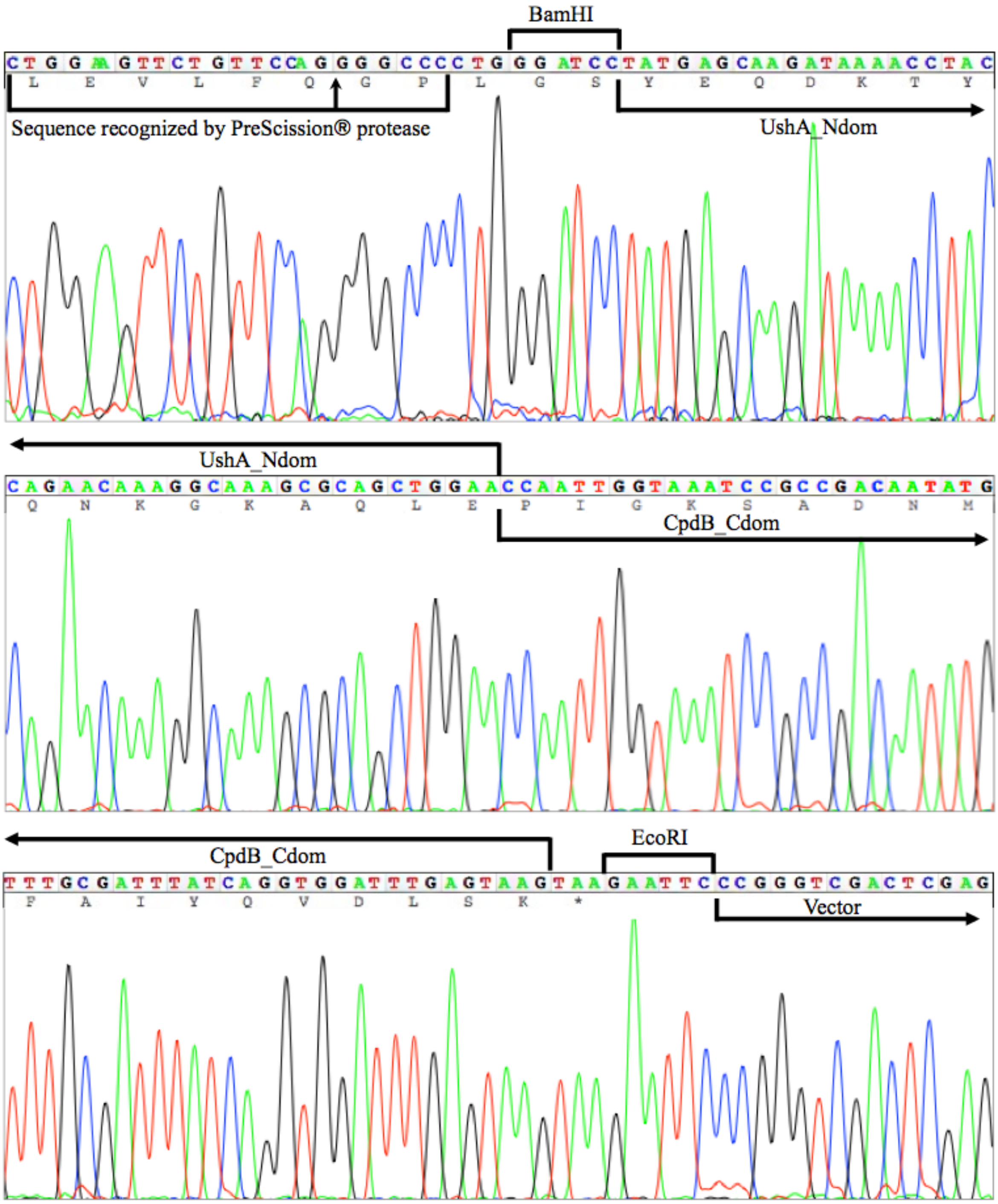

Figure S1. Sanger sequencing of the pGEX-6P-3-UshA_Ndom–CpdB_Cdom plasmid. **Upper panel**, 5′ end of the UshA_Ndom coding sequence preceded by a BamHI site and the coding sequence of the peptide recognized by Prescission protease with the attacked linkage marked by an arrow. **Central panel**, region around the border between UshA_Ndom and CpdB_Cdom. **Lower panel**, 3′ end of the coding sequence of CpdB_Cdom with an EcoRI site after the stop codon.

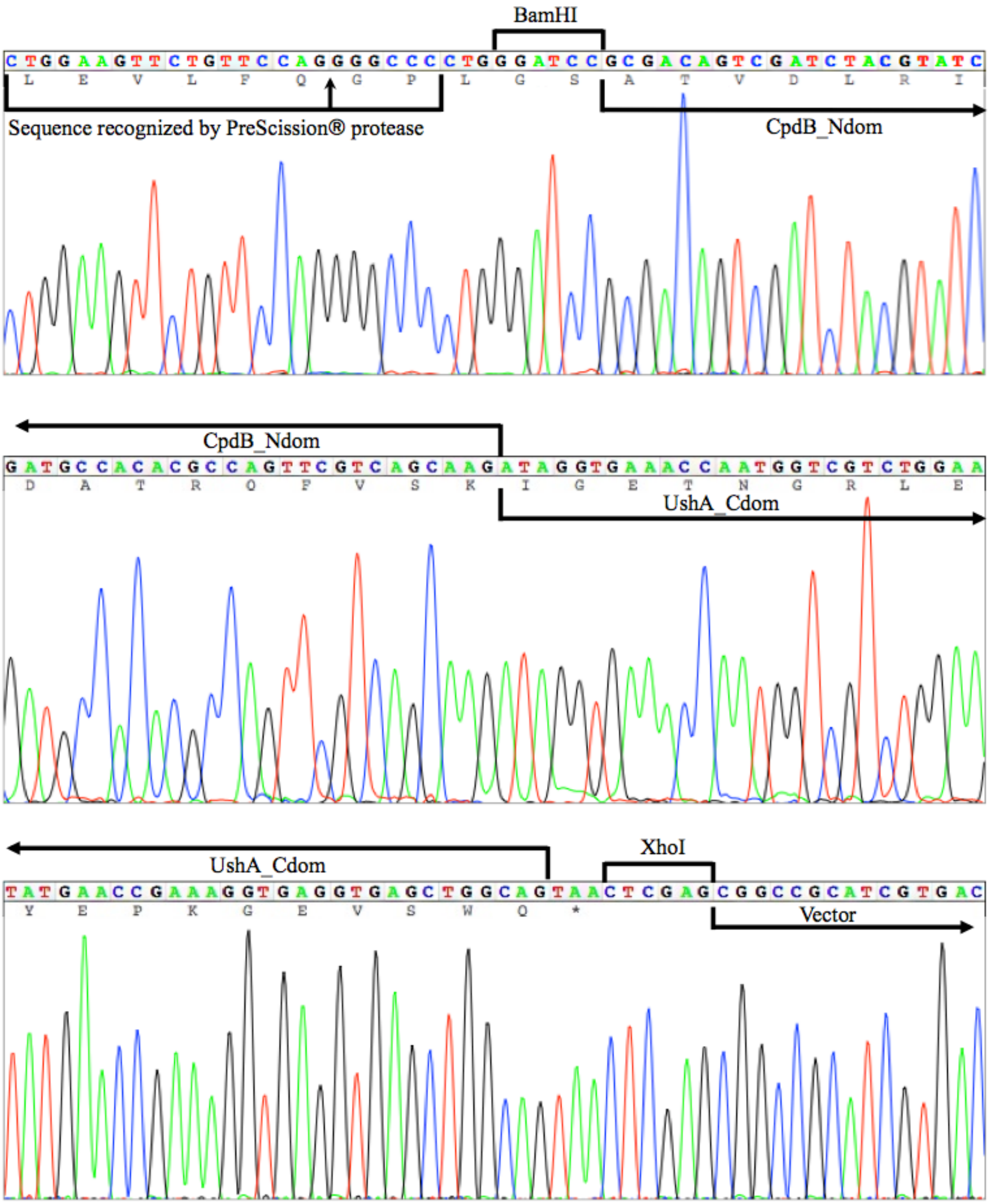

Figure S2. Sanger sequencing of the pGEX-6P-3-CpdB_Ndom–UshA_Cdom plasmid. **Upper panel**, 5′ end of the CpdB_Ndom coding sequence preceded by a BamHI site and the coding sequence of the peptide recognized by Prescission protease with the attacked linkage marked by an arrow. **Central panel**, region around the border between CpdB_Ndom and UshA_Cdom. **Lower panel**, 3′ end of the coding sequence of UshA_Cdom with an XhoI site after the stop codon.

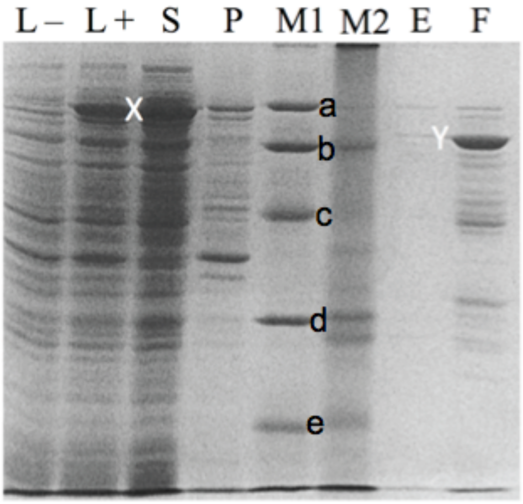

pGEX-6P-3-UshA_Ndom–CpdB_Cdom

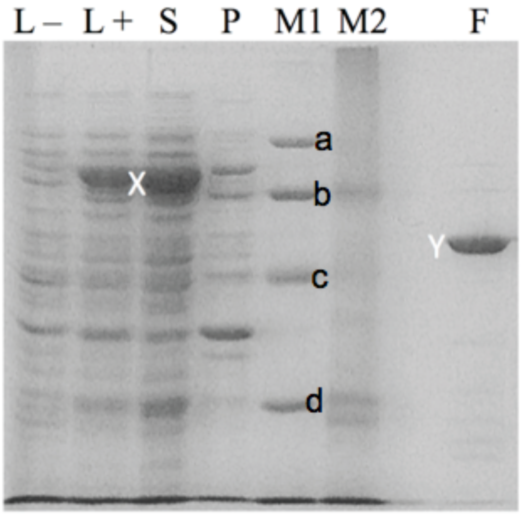

pGEX-6P-3-CpdB_Ndom–UshA_Cdom

Figure S3. Expression of the recombinant proteins from plasmids pGEX-6P-3-UshA_Ndom–CpdB_Cdom and pGEX-6P-3-CpdB_Ndom–UshA_Cdom. L, lysate of BL21 cells transformed with the corresponding plasmid, with (+) or without (–) induction by isopropylthiogalactoside (IPTG). S and P, supernatant and precipitate of the induced cells. M1, molecular weight markers: a, 97.4 kDa; b, 66.2 kDa; c, 45 kDa; d, 31 kDa; e, 21.5 kDa; M2, molecular weight markers (not used). E, fraction excluded from a GSH-Sepharose column after application of induced lysate supernatant. F, fractions collected from the GSH-Sepharose column after in-column removal of the GST tag of fusion proteins with PreScission protease. Letter X marks the position of the GST-fusion proteins with the following expected sizes predicted from their sequences: GST-UshA_Ndom–CpdB_Cdom, 96.8 kDa; GST-CpdB_Ndom–UshA_Cdom, 83.7 kDa. Letter Y marks the recombinant proteins devoid of the GST tag with the following expected sizes predicted from their sequences: GPLGS-UshA_Ndom–CpdB_Cdom, 70.4 kDa; GPLGS-CpdB_Ndom–UshA_Cdom, 57.3 kDa. The Y bands represent 62% (left panel) and 85% (right panel) of the total protein in lanes F.

001 GPLGSYEQDK TYKITVLHTN DHHGHFWRNE YGEYGLAAQK TLVDGIRKEV
051 AAEGGSVLLL SGGDINTGVP ESDLQDAEPD FRGMNLVGYD AMAIGNHEFD
101 NPLTVLRQQE KWAKFPLLSA NIYQKSTGER LFKPWALFKR QDLKIAVIGL
151 TTDDTAKIGN PEYFTDIEFR KPADEAKLVI QELQQTEKPD IIIAATHMGH
201 YDNGEHGSNA PGDVEMARAL PAGSLAMIVG GHSQDTVCMA AENKKQVDYV
251 PGTPCKPDQQ NGIWIVQAHE WGKYVGRADF EFRNGEMKMV NYQLIPVNLK
301 KKVTWEDGKS ERVLYTPEIA ENQQMISLLS PFQNKGKAQL EPIGKSADNM
351 YSYLALVQDD PTVQVVNNAQ KAYVEHYIQG DPDLAKLPVL SAAAPFKVGG
401 RKNDPASYVE VEKGRLTFRN AADLYLYPNT LIVVKASGKE VKEWLECSAG
451 QFNQIDPDNT KPQSLINWDG FRTYNFDVID GVNYQIDVTQ PARYDGECQM
501 VNANAERIKN LTFNGKPIDP NAMFLVATNN YRAYGGKFAG TGDSHIAFAS
551 PDENRSVLAA WIADESKRAG EIHPAADNNW RLAPIAGDKK LDIRFETSPS
601 DKAAAFIKEK GQYPMNKVAT DDIGFAIYQV DLSK

Figure S4. Identification of the recombinant protein GPLGS-UshA_Ndom–CpdB_Cdom by its peptide mass fingerprint. The figure shows the full amino acid sequence obtained by theoretical translation of the plasmid coding sequence. The underlined sequence corresponds to the sum of the experimentally-obtained tryptic peptides that, by MALDI-TOF spectrometry, gave mass signals which matched within 100 ppm error a theoretical prediction performed with PeptideMap (see Appendix S1). Blue, UshA_Ndom sequence. Red, CpdB_Cdom sequence.

001 GPLGSATVDL RIMETTDLHS NMMDFDYYKD TATEKFGLVR TASLINDARN
051 EVKNSVLVDN GDLIQGSPLA DYISAKGLKA GDVHPVYKAL NTLDYTVGTL
101 GNHEFNYGLD YLKNALAGAK FPYVNANVID ARTKQPMFTP YLIKDTEVVD
151 KDGKKQTLKI GYIGVVPPQI MGWDKANLSG KVTVNDITET VRKYVPEMRE
201 KGADVVVVLA HSGLSADPYK VMAENSVYYL SEIPGVNAIM FGHAHAVFPG
251 KDFADIEGAD IAKGTLNGVP AVMPGMWGDH LGVVDLQLSN NSGKWQVTQA
301 KAEARPIYDI ANKKSLAAED SKLVETLKAD HDATRQFVSK IGETNGRLEG
351 DRDKVRFVQT NMGRLILAAQ MDRTGADFAV MSGGGIRDSI EAGDISYKNV
401 LKVQPFGNVV VYADMTGKEV IDYLTAVAQM KPDSGAYPQF ANVSFVAKDG
451 KLNDLKIKGE PVDPAKTYRM ATLNFNATGG DGYPRLDNKP GYVNTGFIDA
501 EVLKAYIQKS SPLDVSVYEP KGEVSWQ

Figure S5. Identification of the recombinant protein GPLGS-CpdB_Ndom–UshA_Cdom by its peptide mass fingerprint. The figure shows the full amino acid sequence obtained by theoretical translation of the plasmid coding sequence. The underlined sequence corresponds to the sum of the experimentally-obtained tryptic peptides that, by MALDI-TOF spectrometry, gave mass signals which matched within 100 ppm error a theoretical prediction performed with PeptideMap (see Appendix S2). Red, CpdB_Ndom sequence. Blue, UshA_Cdom sequence.

Appendix S1. PeptideMap report on the peptide mass fingerprint of the recombinant protein GPLGS-UshA_Ndom–CpdB_Cdom.

PeptideMap - Results

Name: **GPLGS-UshA_Ndom–CpdB_Cdom**

Number of Measured Peptides : 208
Number of Matched Peptides : 53
Min. Coverage of Sequence : 87%

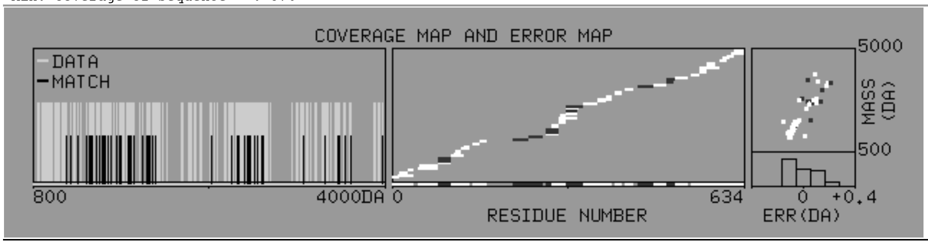

Measured Mass	Ave/ Mono	Computed Mass	Error (Da)	Residues Start	Residues End	Missed Cut	Peptide Sequence
1112.557	M	1112.659	-0.102	387	397	0	LPVLSAAAPFK
1148.563	M	1148.674	-0.111	131	139	0	LFKPWALFK
1205.580	M	1205.567	0.013	303	312	1	VTWEDGKSER
1288.572	M	1288.666	-0.094	556	567	0	SVLAAWIADESK
1292.598	M	1292.712	-0.114	115	125	0	FPLLSANIYQK
1316.596	M	1316.718	-0.122	145	157	0	IAVIGLTTDDTAK
1333.564	M	1333.662	-0.098	302	312	2	KVTWEDGKSER
1341.514	M	1341.620	-0.106	29	40	0	NEYGEYGLAAQK
1377.568	M	1377.677	-0.109	402	413	1	KNDPASYVEVEK
1406.607	M	1406.704	-0.097	591	602	1	LDIRFETSPSDK
1430.704	M	1430.795	-0.091	289	300	0	MVNYQLIPVNLK
1444.689	M	1444.767	-0.078	556	568	1	SVLAAWIADESKR
1449.585	M	1449.674	-0.089	569	581	0	AGEIHPAADNNWR
1461.683	M	1461.757	-0.074	301	312	3	KKVTWEDGKSER
1484.632	M	1484.714	-0.082	1	13	1	GPLGSYEQDKTYK
1510.677	M	1510.766	-0.089	595	608	1	FETSPSDKAAAFIK
1558.799	M	1558.890	-0.091	289	301	1	MVNYQLIPVNLKK
1590.735	M	1590.800	-0.065	402	415	2	KNDPASYVEVEKGR
1599.686	M	1599.756	-0.070	158	170	0	IGNPEYFTDIEFR
1605.701	M	1605.776	-0.075	568	581	1	RAGEIHPAADNNWR
1669.611	M	1669.682	-0.071	494	507	0	YDGECQMVNANAER (1)+C3H5ON@C
1677.849	M	1677.924	-0.075	112	125	1	WAKFPLLSANIYQK
1694.815	M	1694.881	-0.066	603	617	2	AAAFIKEKGQYPMNK
1717.782	M	1717.831	-0.049	372	386	0	AYVEHYIQGDPDLAK
1805.931	M	1805.992	-0.061	420	435	0	NAADLYLYPNTLIVVK
1834.855	M	1835.020	-0.165	126	140	2	STGERLFKPWALFKR
1853.894	M	1853.941	-0.047	618	634	0	VATDDIGFAIYQVDLSK
1868.876	M	1868.918	-0.042	14	28	0	ITVLHTNDHHGHFWR
1890.820	M	1890.849	-0.029	538	555	0	FAGTGDSHIAFASPDENR
1910.926	M	1910.861	0.065	494	509	1	YDGECQMVNANAERIK (1)+C3H5ON@C
2427.195	M	2427.170	0.025	473	493	0	TYNFDVIDGVNYQIDVTQPAR
2609.364	M	2609.306	0.058	510	532	0	NLTFNGKPIDPNAMFLVATNNYR
2662.437	M	2662.367	0.070	313	335	0	VLYTPEIAENQQMISLLSPFQNK
2672.422	M	2672.315	0.107	611	634	1	GQYPMNKVATDDIGFAIYQVDLSK
2678.441	M	2678.362	0.079	313	335	0	VLYTPEIAENQQMISLLSPFQNK (1)+O@M
2721.469	M	2721.423	0.046	108	130	3	QQEKWAKFPLLSANIYQKSTGER
2746.386	M	2746.320	0.066	83	107	0	GMNLVGYDAMAIGNHEFDNPLTVLR
2755.428	M	2755.382	0.046	278	300	2	ADFEFRNGEMKMVNYQLIPVNLK
2762.414	M	2762.315	0.099	83	107	0	GMNLVGYDAMAIGNHEFDNPLTVLR (1)+O@M
2769.440	M	2769.361	0.079	219	245	1	ALPAGSLAMIVGGHSQDTVCMAAENKK (1)+C3H5ON@C
2771.417	M	2771.377	0.040	278	300	2	ADFEFRNGEMKMVNYQLIPVNLK (1)+O@M
2778.455	M	2778.310	0.145	83	107	0	GMNLVGYDAMAIGNHEFDNPLTVLR (2)+O@M
2812.570	M	2812.479	0.091	372	397	1	AYVEHYIQGDPDLAKLPVLSAAAPFK
2847.452	M	2847.484	-0.032	313	337	1	VLYTPEIAENQQMISLLSPFQNKGK

(continued in next page)

2882.483	M	2882.375	0.108	346	371	0	SADNMYSYLALVQDDPTVQV VNNAQK
2899.465	M	2899.472	-0.007	278	301	3	ADFEFRNGEMKMVNYQLIPV NLKK (1)+O@M
3263.746	M	3263.582	0.164	246	273	0	QVDYVPGTPCKPDQQNGIWI VQAHEWGK (1)+C3H5ON@C
3456.843	M	3456.653	0.190	49	82	0	EVAAEGGSVLLLSGGDINTG VPESDLQDAEPDFR
3578.878	M	3578.652	0.226	443	472	0	EWLECSAGQFNQIDPDNTKP QSLINWDGFR (1)+C3H5ON@C
3584.958	M	3584.733	0.225	11	40	2	TYKITVLHTNDHHGHFWRNE YGEYGLAAQK
3584.958	M	3584.748	0.210	48	82	1	KEVAAEGGSVLLLSGGDINT GVPESDLQDAEPDFR
3641.990	M	3641.870	0.120	556	589	3	SVLAAWIADESKRAGEIHPA ADNNWRLAPIAGDK
3699.996	M	3699.954	0.042	313	345	2	VLYTPEIAENQQMISLLSPF QNKGKAQLEPIGK (1)+O@M
3978.123	M	3978.019	0.104	302	335	3	KVTWEDGKSERVLYTPEIAE NQQMISLLSPFQNK

Unmatched Monoisotopic Masses:

849.133 853.247 854.345 858.112 859.176 859.917 882.421 890.305 893.284 900.403 906.382 926.349 927.230
928.243 929.238 934.332 945.282 961.350 980.134 1003.368 1019.388 1021.355 1033.000 1034.010 1036.427 1045.388
1073.428 1075.386 1078.398 1089.467 1090.403 1093.374 1095.411 1096.450 1098.429 1106.424 1170.559 1180.546
1183.468 1206.585 1238.580 1349.623 1361.684 1382.692 1390.580 1398.536 1448.694 1466.584 1476.671 1490.664
1501.715 1506.611 1541.659 1607.626 1621.630 1625.699 1655.602 1656.705 1712.656 1726.649 1774.798 1862.971
1886.886 1900.873 1925.921 1947.843 1951.050 1957.910 1960.908 1982.945 2021.128 2154.062 2162.042 2219.070
2225.165 2243.092 2272.141 2282.190 2318.067 2330.207 2410.207 2424.162 2429.015 2450.211 2484.249 2542.277
2546.252 2554.118 2562.339 2581.323 2594.321 2610.371 2614.399 2626.344 2650.379 2654.374 2667.395 2671.404
2682.425 2683.414 2684.403 2698.393 2707.420 2714.376 2719.446 2722.433 2724.425 2742.419 2766.428 2786.444
2790.411 2798.417 2803.435 2820.445 2827.479 2834.490 2851.561 2860.444 2869.606 2892.508 2908.590 2917.495
2926.581 2939.519 2955.597 3153.613 3193.673 3212.629 3232.691 3233.707 3247.691 3249.727 3250.709 3264.730
3290.709 3306.727 3321.756 3365.759 3378.810 3437.829 3440.862 3451.850 3474.821 3479.832 3513.873 3547.859
3563.830 3564.822 3583.853 3621.843 3679.913 3905.106 3921.090 3936.140 3955.090

Input Summary

Protein

```
      1  *    10   *    20   *    30   *    40   *    50
    1 GPLGSYEQDKTYKITVLHTNDHHGHFWRNEYGEYGLAAQKTLVDGIRKEV
   51 AAEGGSVLLLSGGDINTGVPESDLQDAEPDFRGMNLVGYDAMAIGNHEFD
  101 NPLTVLRQQEKWAKFPLLSANIYQKSTGERLFKPWALFKRQDLKIAVIGL
  151 TTDDTAKIGNPEYFTDIEFRKPADEAKLVIQELQQTEKPDIIIAATHMGH
  201 YDNGEHGSNAPGDVEMARALPAGSLAMIVGGHSQDTVCMAAENKKQVDYV
  251 PGTPCKPDQQNGIWIVQAHEWGKYVGRADFEFRNGEMKMVNYQLIPVNLK
  301 KKVTWEDGKSERVLYTPEIAENQQMISLLSPFQNKGKAQLEPIGKSADNM
  351 YSYLALVQDDPTVQVVNNAQKAYVEHYIQGDPDLAKLPVLSAAAPFKVGG
  401 RKNDPASYVEVEKGRLTFRNAADLYLYPNTLIVVKASGKEVKEWLECSAG
  451 QFNQIDPDNTKPQSLINWDGFRTYNFDVIDGVNYQIDVTQPARYDGECQM
  501 VNANAERIKNLTFNGKPIDPNAMFLVATNNYRAYGGKFAGTGDSHIAFAS
  551 PDENRSVLAAWIADESKRAGEIHPAADNNWRLAPIAGDKKLDIRFETSPS
  601 DKAAAFIKEKGQYPMNKVATDDIGFAIYQVDLSK
```

Enzyme	Name	Site(s)	Terminal	Mod.	Proline	Missed Cut
	Trypsin	KR	C		N	3

Global Modification	Name	Site(s)	Formula	Min. Occ.	Max. Occ.
	Acrylamide (Cys)	C	+C3H5ON	Complete	
	Oxidation (M)	M	+O	Partial	

Monoisotopic Masses (+/- 100 ppm)

849.133 853.247 854.345 858.112 859.176 859.917 882.421 890.305
893.284 900.403 906.382 926.349 927.230 928.243 929.238 934.332
945.282 961.350 980.134 1003.368 1019.388 1021.355 1033.000 1034.010
1036.427 1045.388 1073.428 1075.386 1078.398 1089.467 1090.403
1093.374 1095.411 1096.450 1098.429 1106.424 1112.557 1148.563
1170.559 1180.546 1183.468 1205.580 1206.585 1238.580 1288.572
1292.598 1316.596 1333.564 1341.514 1349.623 1361.684 1377.568
1382.692 1390.580 1398.536 1406.607 1430.704 1444.689 1448.694

(continued in next page)

	1449.585 1461.683 1466.584 1476.671 1484.632 1490.664 1501.715 1506.611 1510.677 1541.659 1558.799 1590.735 1599.686 1605.701 1607.626 1621.630 1625.699 1655.602 1656.705 1669.611 1677.849 1694.815 1712.656 1717.782 1726.649 1774.798 1805.931 1834.855 1853.894 1862.971 1868.876 1886.886 1890.820 1900.873 1910.926 1925.921 1947.843 1951.050 1957.910 1960.908 1982.945 2021.128 2154.062 2162.042 2219.070 2225.165 2243.092 2272.141 2282.190 2318.067 2330.207 2410.207 2424.162 2427.195 2429.015 2450.211 2484.249 2542.277 2546.252 2554.118 2562.339 2581.323 2594.321 2609.364 2610.371 2614.399 2626.344 2650.379 2654.374 2662.437 2667.395 2671.404 2672.422 2678.441 2682.425 2683.414 2684.403 2698.393 2707.420 2714.376 2719.446 2721.469 2722.433 2724.425 2742.419 2746.386 2755.428 2762.414 2766.428 2769.440 2771.417 2778.455 2786.444 2790.411 2798.417 2803.435 2812.570 2820.445 2827.479 2834.490 2847.452 2851.561 2860.444 2869.606 2882.483 2892.508 2899.465 2908.590 2917.495 2926.581 2939.519 2955.597 3153.613 3193.673 3212.629 3232.691 3233.707 3247.691 3249.727 3250.709 3263.746 3264.730 3290.709 3306.727 3321.756 3365.759 3378.810 3437.829 3440.862 3451.850 3456.843 3474.821 3479.832 3513.873 3547.859 3563.830 3564.822 3578.878 3583.853 3584.958 3621.843 3641.990 3679.913 3699.996 3905.106 3921.090 3936.140 3955.090 3978.123
Charge State	MH+

Appendix S2. PeptideMap report on the peptide mass fingerprint of the recombinant protein GPLGS-CpdB_Ndom–UshA_Cdom.

PeptideMap - Results

Name: **GPLGS-CpdB_Ndom–UshA_Cdom**

Number of Measured Peptides : 145
Number of Matched Peptides : 40
Min. Coverage of Sequence : 74%

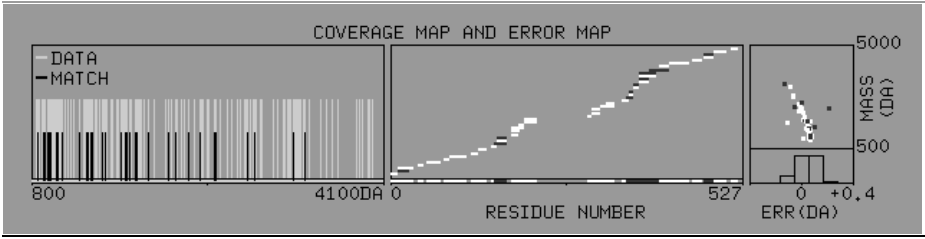

Measured Mass	Ave/ Mono	Computed Mass	Error (Da)	Residues Start	End	Missed Cut	Peptide Sequence
859.527	M	859.455	0.072	295	301	0	WQVTQAK
916.548	M	916.534	0.014	152	159	2	DGKKQTLK
921.550	M	921.474	0.076	193	199	1	KYVPEMR
951.528	M	951.459	0.069	357	364	0	FVQTNMGR
959.579	M	959.503	0.076	41	49	0	TASLINDAR
967.530	M	967.454	0.076	357	364	0	FVQTNMGR (1)+O@M
984.592	M	984.502	0.090	80	88	0	AGDVHPVYK
1029.647	M	1029.564	0.083	365	373	0	LILAAQMDR
1045.632	M	1045.559	0.073	365	373	0	LILAAQMDR (1)+O@M
1084.652	M	1084.587	0.065	1	11	0	GPLGSATVDLR
1086.661	M	1086.578	0.083	348	356	2	LEGDRDKVR
1245.724	M	1245.656	0.068	182	192	0	VTVNDITETVR
1319.714	M	1319.660	0.054	510	521	0	SSPLDVSVYEPK
1337.712	M	1337.639	0.073	374	387	0	TGADFAVMSGGGIR
1353.701	M	1353.634	0.067	374	387	0	TGADFAVMSGGGIR (1)+O@M
1359.778	M	1359.714	0.064	302	313	0	AEARPIYDIANK
1373.821	M	1373.751	0.070	182	193	1	VTVNDITETVRK
1377.748	M	1377.704	0.044	121	132	0	FPYVNANVIDAR
1465.855	M	1465.745	0.110	353	364	2	DKVRFVQTNMGR (1)+O@M
1465.855	M	1465.800	0.055	133	144	1	TKQPMFTPYLIK
1467.836	M	1467.768	0.068	323	335	1	LVETLKADHDATR
1487.857	M	1487.809	0.048	302	314	1	AEARPIYDIANKK
1635.808	M	1635.919	-0.111	452	466	2	LNDLKIKGEPVDPAK
1683.810	M	1683.767	0.043	470	485	0	MATLNFNATGGDGYPR
1699.812	M	1699.762	0.050	470	485	0	MATLNFNATGGDGYPR (1)+O@M
1723.932	M	1723.860	0.072	403	418	0	VQPFGNVVVYADMTGK
1771.956	M	1771.933	0.023	160	175	0	IGYIGVVPPQIMGWDK
1787.955	M	1787.928	0.027	160	175	0	IGYIGVVPPQIMGWDK (1)+O@M
1897.019	M	1896.994	0.025	202	220	0	GADVVVVLAHSGLSADPYK
2092.099	M	2092.084	0.015	486	504	0	LDNKPGYVNTGFIDAEVLK
2149.103	M	2149.120	-0.017	182	199	2	VTVNDITETVRKYVPEMR
2154.129	M	2154.132	-0.003	200	220	1	EKGADVVVVLAHSGLSADPYK
2269.163	M	2268.937	0.226	12	29	0	IMETTDLHSNMMDFDYYK (1)+O@M
2269.163	M	2269.155	0.008	315	335	2	SLAAEDSKLVETLKADHDATR
2386.255	M	2386.308	-0.053	155	175	2	KQTLKIGYIGVVPPQIMGWDK (1)+O@M
2388.210	M	2388.217	-0.007	54	76	0	NSVLVDNGDLIQGSPLADYISAK
2516.164	M	2516.185	-0.021	374	398	1	TGADFAVMSGGGIRDSIEAGDISYK
2532.184	M	2532.180	0.004	374	398	1	TGADFAVMSGGGIRDSIEAGDISYK (1)+O@M
2830.313	M	2830.381	-0.068	89	113	0	ALNTLDYTVGTLGNHEFNYGLDYLK
2898.176	M	2898.239	-0.063	12	35	1	IMETTDLHSNMMDFDYYKDTATEK
3258.535	M	3258.627	-0.092	419	448	0	EVIDYLTAVAQMKPDSGAYPQFANVSFVAK
3372.553	M	3372.691	-0.138	388	418	2	DSIEAGDISYKNVLKVQPFGNVVVYADMTGK (1)+O@M

(continued in next page)

Unmatched Monoisotopic Masses:
850.471 873.556 903.546 930.582 960.571 978.574 981.638 989.525 994.547 1008.557 1016.610 1022.626 1038.656 1041.594 1063.607 1070.606 1111.603 1120.633 1141.671 1168.621 1198.698 1289.716 1302.749 1346.733 1376.743 1394.733 1400.763 1416.799 1417.842 1434.791 1474.849 1491.801 1503.642 1544.884 1560.677 1601.900 1608.781 1657.917 1675.887 1681.815 1692.840 1727.819 1732.915 1740.817 1780.959 1797.859 1814.971 1828.976 1862.962 1954.040 2060.096 2114.061 2117.119 2118.111 2162.050 2204.937 2206.122 2211.156 2236.149 2273.152 2327.177 2387.278 2389.203 2413.183 2444.278 2446.226 2468.165 2573.174 2633.381 2681.358 2739.394 2753.394 2802.220 2813.408 2850.193 2887.349 2907.196 2944.353 2955.185 3067.461 3115.478 3163.488 3172.496 3210.539 3220.487 3230.485 3267.571 3275.528 3280.497 3299.563 3315.564 3325.567 3348.538 3358.554 3405.556 3513.626 3568.680 3615.681 3674.659 3853.769 3879.797 3910.762 3957.865 3969.763 4005.874

Input Summary

Protein

```
      1  *    10    *    20    *    30    *    40    *    50
  1 GPLGSATVDLRIMETTDLHSNMMDFDYYKDTATEKFGLVRTASLINDARN
 51 EVKNSVLVDNGDLIQGSPLADYISAKGLKAGDVHPVYKALNTLDYTVGTL
101 GNHEFNYGLDYLKNALAGAKFPYVNANVIDARTKQPMFTPYLIKDTEVVD
151 KDGKKQTLKIGYIGVVPPQIMGWDKANLSGKVTVNDITETVRKYVPEMRE
201 KGADVVVVLAHSGLSADPYKVMAENSVYYLSEIPGVNAIMFGHAHAVFPG
251 KDFADIEGADIAKGTLNGVPAVMPGMWGDHLGVVDLQLSNNSGKWQVTQA
301 KAEARPIYDIANKKSLAAEDSKLVETLKADHDATRQFVSKIGETNGRLEG
351 DRDKVRFVQTNMGRLILAAQMDRTGADFAVMSGGGIRDSIEAGDISYKNV
401 LKVQPFGNVVVYADMTGKEVIDYLTAVAQMKPDSGAYPQFANVSFVAKDG
451 KLNDLKIKGEPVDPAKTYRMATLNFNATGGDGYPRLDNKPGYVNTGFIDA
501 EVLKAYIQKSSPLDVSVYEPKGEVSWQ
```

Enzyme

Name	Site(s)	Terminal	Mod.	Proline	Missed Cut
Trypsin	KR	C		N	3

Global Modification

Name	Site(s)	Formula	Min. Occ.	Max. Occ.
Acrylamide (Cys)	C	+C3H5ON	Complete	
Oxidation (M)	M	+O	Partial	

Monoisotopic Masses (+/- 100 ppm)

850.471 859.527 873.556 903.546 916.548 921.550 930.582 951.528 959.579 960.571 967.530 978.574 981.638 984.592 989.525 994.547 1008.557 1016.610 1022.626 1029.647 1038.656 1041.594 1045.632 1063.607 1070.606 1084.652 1086.661 1111.603 1120.633 1141.671 1168.621 1198.698 1245.724 1289.716 1302.749 1319.714 1337.712 1346.733 1353.701 1359.778 1373.821 1376.743 1377.748 1394.733 1400.763 1416.799 1417.842 1434.791 1465.855 1467.836 1474.849 1487.857 1491.801 1503.642 1544.884 1560.677 1601.900 1608.781 1635.808 1657.917 1675.887 1681.815 1683.810 1692.840 1699.812 1723.932 1727.819 1732.915 1740.817 1771.956 1780.959 1787.955 1797.859 1814.971 1828.976 1862.962 1897.019 1954.040 2060.096 2092.099 2114.061 2117.119 2118.111 2149.103 2154.129 2162.050 2204.937 2206.122 2211.156 2236.149 2269.163 2273.152 2327.177 2386.255 2387.278 2388.210 2389.203 2413.183 2444.278 2446.226 2468.165 2516.164 2532.184 2573.174 2633.381 2681.358 2739.394 2753.394 2802.220 2813.408 2830.313 2850.193 2887.349 2898.176 2907.196 2944.353 2955.185 3067.461 3115.478 3163.488 3172.496 3210.539 3220.487 3230.485 3258.535 3267.571 3275.528 3280.497 3299.563 3315.564 3325.567 3348.538 3358.554 3372.553 3405.556 3513.626 3568.680 3615.681 3674.659 3853.769 3879.797 3910.762 3957.865 3969.763 4005.874

Charge State MH+
